# Transcriptional Modulation of Human Endogenous Retroviruses in Primary CD4+ T Cells Following Vorinostat Treatment

**DOI:** 10.3389/fimmu.2018.00603

**Published:** 2018-04-12

**Authors:** Cory H. White, Nadejda Beliakova-Bethell, Steven M. Lada, Michael S. Breen, Tara P. Hurst, Celsa A. Spina, Douglas D. Richman, John Frater, Gkikas Magiorkinis, Christopher H. Woelk

**Affiliations:** ^1^Faculty of Medicine, University of Southampton, Southampton, Hants, United Kingdom; ^2^San Diego VA Medical Center and Veterans Medical Research Foundation, San Diego, CA, United States; ^3^Department of Medicine, University of California San Diego, La Jolla, CA, United States; ^4^Department of Genetic and Genomic Sciences, Icahn School of Medicine at Mount Sinai, New York, NY, United States; ^5^Department of Zoology, University of Oxford, Oxford, United Kingdom; ^6^Department of Pathology, University of California San Diego, La Jolla, CA, United States; ^7^Nuffield Department of Clinical Medicine, Peter Medawar Building for Pathogen Research, South Parks Road, Oxford, United Kingdom

**Keywords:** human endogenous retroviruses, histone deacetylase inhibitor, primary CD4+ T cells, total RNA-Seq, long terminal repeat

## Abstract

The greatest obstacle to a cure for HIV is the provirus that integrates into the genome of the infected cell and persists despite antiretroviral therapy. A “shock and kill” approach has been proposed as a strategy for an HIV cure whereby drugs and compounds referred to as latency-reversing agents (LRAs) are used to “shock” the silent provirus into active replication to permit “killing” by virus-induced pathology or immune recognition. The LRA most utilized to date in clinical trials has been the histone deacetylase (HDAC) inhibitor—vorinostat. Potentially, pathological off-target effects of vorinostat may result from the activation of human endogenous retroviruses (HERVs), which share common ancestry with exogenous retroviruses including HIV. To explore the effects of HDAC inhibition on HERV transcription, an unbiased pharmacogenomics approach (total RNA-Seq) was used to evaluate HERV expression following the exposure of primary CD4^+^ T cells to a high dose of vorinostat. Over 2,000 individual HERV elements were found to be significantly modulated by vorinostat, whereby elements belonging to the ERVL family (e.g., LTR16C and LTR33) were predominantly downregulated, in contrast to LTR12 elements of the HERV-9 family, which exhibited the greatest signal, with the upregulation of 140 distinct elements. The modulation of three different LTR12 elements by vorinostat was confirmed by droplet digital PCR along a dose–response curve. The monitoring of LTR12 expression during clinical trials with vorinostat may be indicated to assess the impact of this HERV on the human genome and host immunity.

## Introduction

Vorinostat is a histone deacetylase (HDAC) inhibitor also known as suberoylanilide hydroxamic acid. HDAC inhibitors act on HDAC enzymes and block the removal of acetyl groups from histones resulting in a relaxed chromatin state (1) and the modulation of the expression of large numbers of genes (2, 3). In addition, HDAC inhibitors appear to affect the acetylation states of transcription factors at the protein level, which alters their activity and leads to further transcriptional changes (4). HDAC inhibitors have wide ranging therapeutic value and have been considered for the treatment of cancer (5) and neurodegenerative disorders (6), as well as in “shock and kill” strategies to facilitate an HIV cure (7). The therapeutic efficacy of HDAC inhibitors against cancer is thought to stem from their ability to induce tumor cell apoptosis (5). Vorinostat is approved by the Federal Drug Administration (FDA) for the treatment of refractory cutaneous T-cell lymphoma (8). In an HIV cure setting, HDAC inhibitors may provide the “shock” capable of flushing HIV out of the persistent reservoir, while antiretroviral therapy is used to prevent new infections so that the cell lysis mediated by viral replication or the immune system may then “kill” actively replicating cells (7). Due to the pre-existing FDA approvals for human use, vorinostat has already been used in a number of completed (9, 10) and ongoing (11) clinical trials assessing shock and kill strategies for an HIV cure.

Human endogenous retroviruses (HERVs), which constitute approximately 8% of the human genome, are themselves descended from ancient exogenous retroviruses (12) and thus share common ancestry with HIV. HERV structure reflects that of retroviruses with two long terminal repeat (LTR) elements flanking *gag, pol*, and *env* genes, although HERVs most frequently exist in the genome as solitary LTR elements due to the loss of genes through recombination (13). Since vorinostat activates the expression of HIV, there have been concerns that this drug may also upregulate HERVs with potentially pathological consequences (14). For example, HERV pathology could result from the modulation of the expression of protein coding genes or the formation of chimeric proteins with aberrant function leading to oncogenesis (15), as well as the dysregulation of inflammatory immune responses through the expression of HERV encoded proteins (e.g., *gag* and *env*) (16). Indeed, HERV expression has previously been associated with a wide repertoire of diseases including diabetes, schizophrenia, autoimmune diseases (e.g., multiple sclerosis and rheumatoid arthritis), and cancer (17). However, the difficulties in associating HERVs with disease should be stressed due to their ubiquitous nature in human populations although polymorphisms between individuals could explain disease specificity (17). Finally, it was previously shown that HIV capsids could be successfully pseudotyped *in vitro* with HERV-W Env resulting in infectious virus particles (18). This raises the possibility that coexpression of HERVs and HIV might lead to novel retroviral strains with new properties through transcomplementation or recombination, although the latter may be unlikely due to the large evolutionary distance between HERV elements and HIV (19).

To explore the ability of vorinostat to modulate the expression of HERV elements in the human genome, our previous analysis utilized a targeted approach [i.e., real-time reverse transcription polymerase chain reaction (RT-qPCR)], to assess the expression of the *env* and *pol* genes of specific HERV families (i.e., HERV-K, HERV-W, and HERV-FRD) following HDAC inhibitor treatment (14). This study showed that cell line model systems of chronic HIV infection (i.e., J-LAT-8.4 and U1 cells) treated with different concentrations of vorinostat (i.e., 1 µM and 1 mM) for 24 h did not significantly alter the expression of these HERV elements. Furthermore, treatment of uninfected and HIV-infected primary CD4^+^ T cells with another HDAC inhibitor, panobinostat (20 nM), for 24 h did not result in the upregulation of these HERV genes. In contrast, Kronung et al. (20) previously applied another targeted RT-qPCR approach to study the expression of transcripts of the *TP63* and *TNFRSF10B* genes that are under control of an LTR12 promoter derived from the HERV-9 family. Treatment with vorinostat (1 or 5 µM) for 18 h upregulated these genes *via* the LTR12 promoter across various cells lines (i.e., GH, H1299, K562, U2OS, HeLa, Ovcar-3, and HuT-78) suggesting that this drug may indeed modulate HERV elements. However, discrepancies have been noted between cell lines and primary cells with respect to the host gene transcriptional profile induced by vorinostat (2). The main motivation for the current study was to resolve these discrepancies and determine if vorinostat can modulate HERV elements in primary CD4^+^ T cells using an unbiased approach (i.e., total RNA-Seq). Uninfected instead of HIV-infected primary CD4^+^ T cells were selected for study to disambiguate the effects of vorinostat on HERV elements since the Tat protein of HIV has also been shown to activate HERV elements, e.g., HERV-K(HML-2) (21, 22).

## Materials and Methods

### Isolation of Primary CD4+ T Cells

For subsequent total RNA-Seq analysis, cryopreserved primary CD4^+^ T cells that were viably frozen were obtained from four different healthy donors (AllCells, Inc., Emeryville, CA, USA) and thawed in RPMI with 20% human serum. Dead cells resulting from thawing frozen cells were removed using Viahance magnetic negative selection (Biophysics Assay Laboratory Inc., Worcester, MA, USA). For dose–response analysis, peripheral blood was isolated from two additional healthy donors by venipuncture according to the protocols approved by an institutional review board into polypropylene syringes containing sodium heparin. Primary CD4^+^ T cells were isolated using the RosetteSep CD4^+^ T cell enrichment cocktail (StemCell Technologies Inc., Vancouver, Canada). Aliquots taken from CD4^+^ T cell samples were subjected to flow cytometry to assess purity (i.e., >95% cells expressing CD4).

### Treatment of Primary CD4+ T Cells With Vorinostat

Primary CD4^+^ T cells (2.5 million cells per milliliter) were plated into six-well tissue culture plates at 2 ml per well. For the four donors subjected to total RNA-Seq analysis, wells were either treated with a high dose of vorinostat (10 µM) dissolved in dimethyl sulfoxide (DMSO) or left untreated (i.e., DMSO solvent only). For the two donors subjected to dose–response analysis by digital droplet PCR, the wells were treated with 0.34, 1, 3, and 10 µM of vorinostat dissolved in DMSO or left untreated (i.e., DMSO solvent only). In all cases, after 24 h of vorinostat exposure, the samples were washed twice with 10 ml of phosphate buffered saline and resuspended in RLT Plus buffer (Qiagen, Valencia, CA, USA) containing β-mercaptoethanol for RNA extraction.

### RNA Isolation

Total RNA was extracted from primary CD4^+^ T cells using the RNeasy Plus Kit (Qiagen, Valencia, CA, USA) and genomic DNA removed using an on-column DNase treatment. RNA integrity was assessed using the Bioanalyzer 2100 (Agilent Technologies, Santa Clara, CA, USA) and RNA integrity numbers of samples were on average 8.9 (SD ± 0.29).

### Total RNA-Seq Data Generation

Cytoplasmic and mitochondrial ribosomal RNAs (rRNAs) were removed from total RNA extractions using the Ribo-Zero Gold (Human/Mouse/Rat) rRNA Removal Kit (Epicentre, Madison, WI, USA). RNA-Seq libraries were prepared using the TruSeq™ Stranded Total RNA Library Prep Kit (Illumina, San Diego, CA, USA) and sequenced to a depth of 100 million reads using the HiSeq2000 (Illumina) to generate 50 bp paired-end reads.

### Total RNA-Seq Data Analysis

Sequence data in FASTA format for greater than 92,000 distinct HERV elements from the human genome were downloaded from the Endogenous Retrovirus Database (HERVd, 2012 release) (23, 24). This FASTA file of HERV sequence data was converted into a Bowtie index using the *bowtie-build* command (25) and also used to manually construct a gene transfer format (GTF) file. Duplication has led to the expansion of HERV elements throughout the human genome that are often fragmented due to insertions. To enable accurate quantification of HERV elements, reads were mapped with the “-m 1” option using the Bowtie index to ensure that only reads uniquely mapping to a single HERV element with no mismatches were retained. To maximize the alignment of reads to fragments of HERV elements, paired-end reads were decoupled into single-end reads for mapping purposes. The number of reads mapping to each HERV element was then counted using htseq-count with the GTF file (26). Raw counts were converted to counts per million (cpm) mapped reads, HERV elements were removed that did not have at least 1 cpm in at least half of the samples, and subjected to trimmed mean of M-values normalization. Finally, reads from the total RNA-Seq data were also mapped to the human genome using TopHat (27) (default settings with coverage based search for junctions disabled) in order to visualize read pile up against HERV elements in a genomic context using the UCSC genome browser (28).

### Total RNA-Seq Data Access

Metadata, FASTQ files, and a raw HERV expression matrix have been submitted to the Gene Expression Omnibus (https://www.ncbi.nlm.nih.gov/geo/) under accession number GSE102187.

### Droplet Digital PCR (ddPCR) Analysis

Custom TaqMan assays were used to quantify the upregulation by vorinostat of three LTR12 elements with HERVd designations rv_007357, rv_007420, and rv_010177, using the QX100 Droplet Digital PCR System (Bio-Rad, Hercules, CA, USA) as previously described (29, 30). Briefly, the PrimerQuest Tool (31) (Integrated DNA Technologies, Coralville, IA, USA) was used to design two TaqMan assays against rv_007357 and a single assay against each of the remaining LTR12 elements. Primer and probe sequences for these TaqMan assays were subject to BLAT analysis against the human genome to confirm specificity and are presented in Table 1. Five nanograms of RNA in a 20 µl PCR reaction volume were used for each target in duplicate. The TaqMan Gene Expression Assay (Hs03044961_g1) for the ribosomal protein L27 (*RPL27*) gene was selected as a normalizer (32). LTR12 element expression was assessed between the vorinostat treated (10 µM) and untreated condition using the samples used to generate the original total RNA-Seq data (Donors 1–4). LTR12 expression was also assessed in a vorinostat dose–response curve (0, 0.34, 1, 3, and 10 µM) for two additional donors (Donors 5 and 6).

**Table 1:** Custom TaqMan assays used to confirm the upregulation of LTR12 elements by vorinostat treatment.^a^

LTR12 element	Primer and probe set	Designation	Sequence	Length	GC%	Amplicon size (bp)
rv_007357	Set 1	Forward (sense)	GAGCGTATGGCGTTATGTAGTT	22	45.5	114
		Probe (sense)	TTGAGCCGATGAGATCGCTAAGCC	24	54.0	
		Reverse (antisense)	AGCGGTATGTCCTCCCTTTA	20	50.0	

	Set 2	Forward (sense)	GGAGGAACGAAACACTCATCT	21	47.6	102
		Probe (antisense)	TGCAACTTTCACAGAGTCGTCTCACC	26	50.0	
		Reverse (antisense)	CGTCTCACCCACTTCAGAAA	20	50.0	

rv_007420	Set 1	Forward (sense)	GGTAGTGAGAGAGAACGGTATG	22	50.0	124
		Probe (sense)	TCCTCTGCTCATTCTGGTTGTGCT	24	50.0	
		Reverse (antisense)	CTAAAGAGCTCCCACGGTATAG	22	50.0	

rv_010177	Set 1	Forward (sense)	ACTCCAGACACACCGTCTTA	20	50.0	96
		Probe (sense)	ATTGGTAGCTTTCCCGAGTCAGCG	24	54.0	
		Reverse (antisense)	TCATTCCATTCAGGTGGGTTC	21	47.6	

*^a^The “rv” designations from the Human Endogenous Retrovirus Database (HERVd) are listed for each LT12 element. Two primer and probe sets were used for the LTR12 element with designation rv_007357*.

*GC%, percentage of guanine and cytosine bases in corresponding primer or probe; bp, base pair*.

### Statistical Analyses

For total RNA-Seq data, HERV elements were identified as differentially expressed between vorinostat treated and untreated samples with a false discovery rate (FDR) corrected *p*-value <0.05 using EdgeR (33). EdgeR adopts the negative binomial model as the main approach to model RNA-Seq data. This model approach requires an estimate of the true biological coefficient of variation. The square root of this value, the dispersion parameter, was estimated in edgeR by initially measuring a single dispersion parameter using all genes, while taking into account trends or gene abundances (i.e., trended dispersion). Then genewise (i.e., tagwise) dispersion estimates are measured and an empirical Bayes method was used to shrink these genewise dispersion estimates toward the trended dispersion. Gene expression data were then fit to the generalized linear model (GLM), and a GLM likelihood test was used to assess differential gene expression. Model parameters included a sample donor variable to account for the paired structure of the data (paired before and after vorinostat treatment). Significance values were adjusted for multiple testing using the Benjamini and Hochberg (34) method to control the FDR.

Validation of gene expression initially assessed by total RNA-Seq was performed using ddPCR, which is more sensitive than RT-qPCR, since target RNA is distributed across thousands of oil emulsion droplets that each undergoes reverse transcription and a subsequent end point PCR reaction. The number of target RNA molecules present was calculated from the fraction of positive end point reactions using Poisson statistics because some droplets contain no template while others contain one or more copies (35). All ddPCR data were expressed as copies of target RNA molecules (e.g., LTR12 element) per million copies of *RPL27* mRNA molecules and then log_2_ transformed. Prior to log_2_ transformation, a small regularization constant of 0.01 was added to all values used in this calculation to avoid taking a logarithm of zero in some instances. Differential expression of LTR12 elements was assessed between the vorinostat treated (10 µM) and untreated condition in a paired *t*-test (*p*-value < 0.05) using the samples used to generate the original total RNA-Seq data (Donors 1–4), as well as along the aforementioned dose–response curve for Donors 5 and 6.

## Results

### Global Modulation of HERVs Upon Treatment With Vorinostat

**Figure 1:**
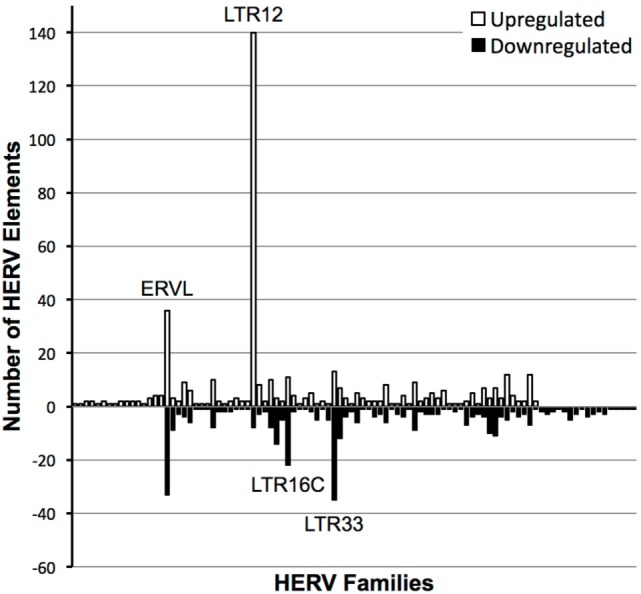
Number of distinct human endogenous retrovirus (HERV) elements per HERV family modulated by vorinostat treatment. HERV elements significantly differentially expressed (false discovery rate-corrected *p*-value < 0.05) with a log_2_ fold change of greater than 3 or less than −3 between vorinostat treated and untreated samples are depicted. HERV elements were grouped into HERV families according to designations in the Human Endogenous Retrovirus Database. White bars represent the number of HERV elements upregulated by vorinostat and black bars the number of downregulated elements. For example, there were 140 distinct HERV elements belonging to the LTR12 family which were significantly upregulated with a log_2_ fold change greater than 3 by vorinostat treatment.

Primary CD4^+^ T cells were isolated from four seronegative donors and treated with a high dose of vorinostat (10 µM) or left untreated for 24 h. This high dose of vorinostat was initially used in an exploratory approach to optimize the ability to identify HERV elements modulated by this HDAC inhibitor. These eight samples were subjected to total RNA-Seq analysis, and the resulting data mapped against HERV sequences curated in HERVd (23). The non-parametric equivalent of a paired *t*-test identified 2,101 distinct HERV elements modulated by vorinostat with an FDR corrected *p*-value of less than 0.05 that mapped to 120 different HERV families across the human genome. In a conservative approach, HERV elements with an absolute log_2_ fold change of more than |3| were identified, leaving 451 upregulated HERV elements from 81 distinct HERV families and 363 downregulated elements from 82 families annotated from HERVd (Figure 1). LTR16C and LTR33 elements, which originated from the ERVL family, were predominantly downregulated, whereas LTR12 elements from the HERV-9 family were predominantly upregulated. The upregulation of LTR12 elements was by far the most dramatic of which the most upregulated (rv_005487) had a log_2_ fold change of 11.985 (actual fold change 4,054). Furthermore, the top 100 upregulated HERV elements contained 46 that were from the LTR12 HERV family (data not shown). Other ERVL elements (i.e., not belonging to the LTR16C or LTR33 families) had a balance of up- and downregulated elements. In summary, vorinostat clearly modulated HERV elements across the genome, but appears to have specificity for certain elements (e.g., LTR12) and families (e.g., ERVL and HERV-9).

Previously, using targeted RT-qPCR analysis we demonstrated that cell lines chronically infected with HIV (i.e., J-LAT8.4 or U1 cells) exposed to low (1 µM) and high (1 mM) doses of vorinostat did not lead to the consistent upregulation of the following HERV elements: HK2 *env*, HK2 *pol*, HERV-W *env* (syncytin-1), and HERV-FRD *env* (syncytin-2) (14). To confirm these findings, the expression of these elements in the total RNA-Seq data of the current study was examined following vorinostat treatment of primary CD4^+^ T cells. There was no difference in expression of these elements between vorinostat treated and untreated cells (Figure S1 in Supplementary Material).

### ddPCR Confirmation of LTR12 Upregulation by Vorinostat

**Figure 2:**
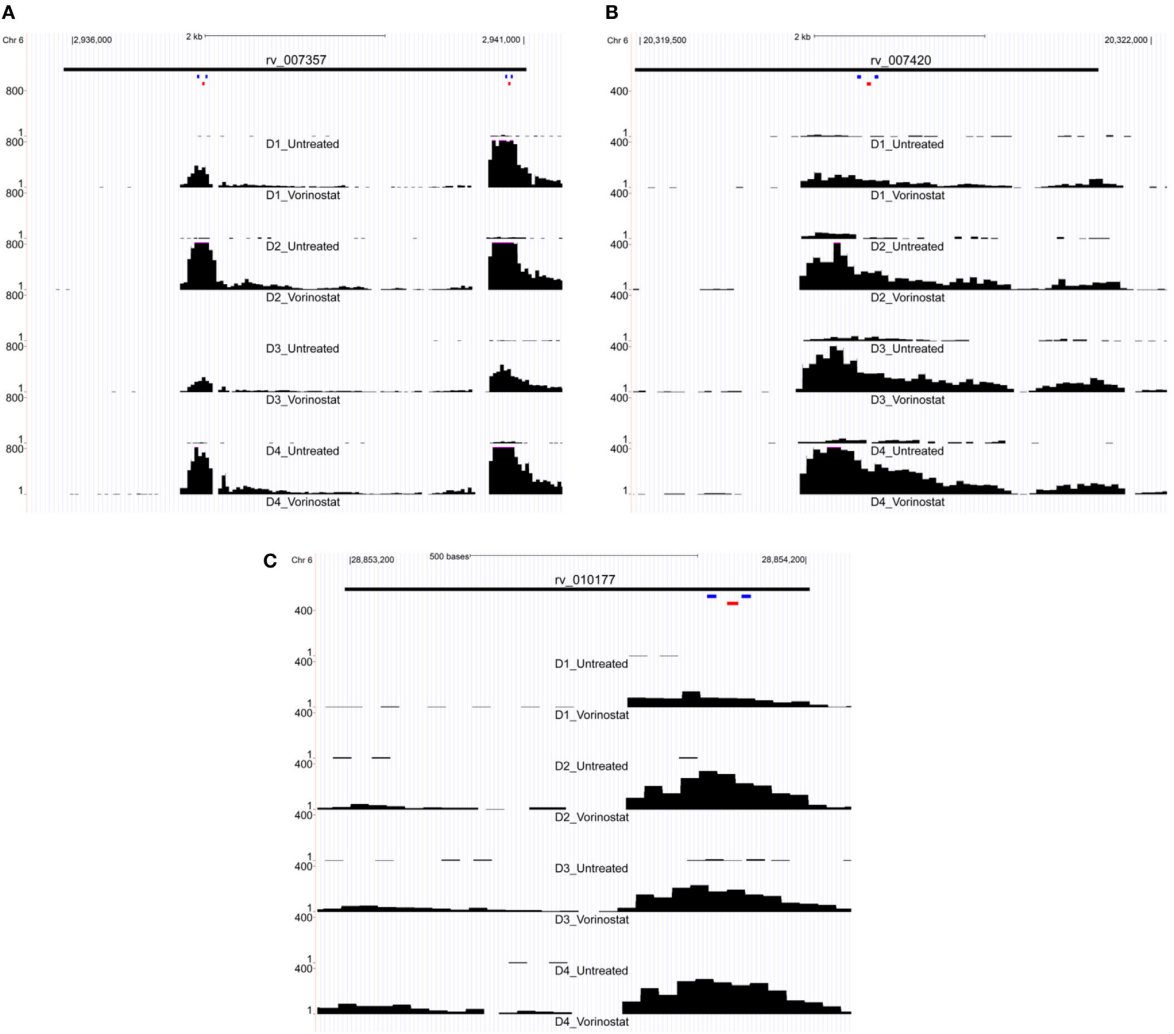
Expression of LTR12 elements visualized as read pile up using the UCSC genome browser. Reads from the total RNA-Seq experiment were mapped to the human genome and then uploaded to the UCSC genome browser for visualization of each LTR12 element: **(A)** rv_007357, **(B)** rv_007420, and **(C)** rv_010177. Read tracts are labeled with the donor and condition, e.g., *D1* for Donor 1, *Vorinostat* for drug treated, and *Untreated* for the untreated control (i.e., dimethyl sulfoxide solvent alone). Chromosomal coordinates are depicted at the top of each figure, and the black bar below indicates the position of the LTR12 element. LTR12 elements are labeled with their “rv” designation from the Human Endogenous Retrovirus Database. The *y*-axis indicates the read level averaged over 40 bp, and the pink caps to black bars in the figure indicate reads whose numbers extended beyond the depicted scale. Small colored bars represent the position of primers (blue) and probes (red) from custom TaqMan assay used to assess the expression of LTR12 elements by droplet digital PCR. Two distinct TaqMan assays were targeted to rv_007357 **(A)** with a single assay against each of the remaining LTR12 elements **(B,C)**.

**Figure 3:**
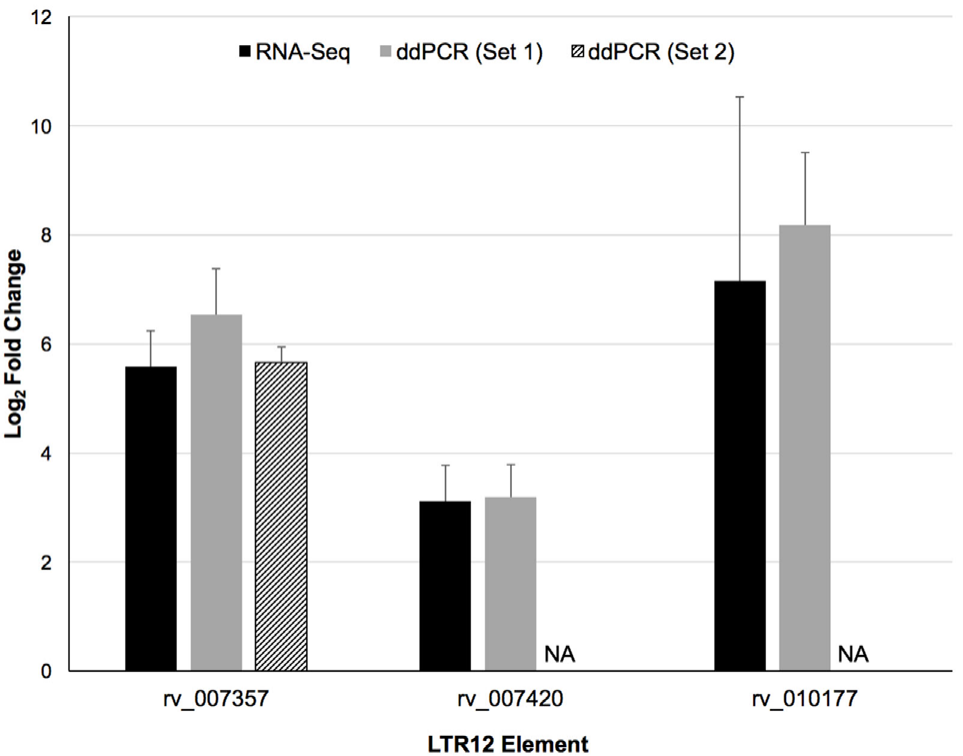
Confirmation of the upregulation of three distinct LTR12 elements by vorinostat in primary CD4^+^ T cells using droplet digital PCR (ddPCR). Log_2_ fold changes between vorinostat treated and untreated conditions were averaged across all four donors (Donors 1–4) for the RNA-Seq (black bars) and the ddPCR (gray and hatched bars) data for each LTR12 element. Error bars represent SDs across donors. The labels “Set 1” and “Set 2” indicate the two distinct primer and probe sets used to target the same LTR12 element (i.e., rv_007357). A second primer and probe set was not used to target the other LTR12 elements, and the missing bar is thus labeled “NA” for not applicable. LTR12 elements are labeled with their “rv” designation from the Human Endogenous Retrovirus Database.

LTR12 elements were selected for ddPCR validation since these elements were the most upregulated by vorinostat. Three LTR12 elements on chromosome 6 (rv_007357, rv_007420, and rv_010177) were selected for ddPCR analysis since they were (1) upregulated by at least eightfold (log_2_ fold change of 3), (2) longer than 1,000 bp and upregulated along large segments of the HERV element, (3) not confounded by the presence of a neighboring gene within 5 kb, and (4) consistently upregulated across all donors (Figure 2). The most upregulated LT12 element (rv_005487) is approximately 1.5 kb in the human genome but was not selected for ddPCR analysis because only a 250 bp fragment of this element was expressed. Two TaqMan primer and probe sets were designed against rv_007357 to capture the two major peaks of expression in this LTR12 element, whereas a single primer and probe set was used to target rv_007420 and rv_010177 (Table 1; Figure 2). Significant upregulation of all three elements upon vorinostat exposure was confirmed by ddPCR and fold changes reflected those detected by RNA-Seq analysis (Figure 3).

### Dose Responsive Upregulation of LTR12 by Vorinostat

**Figure 4:**
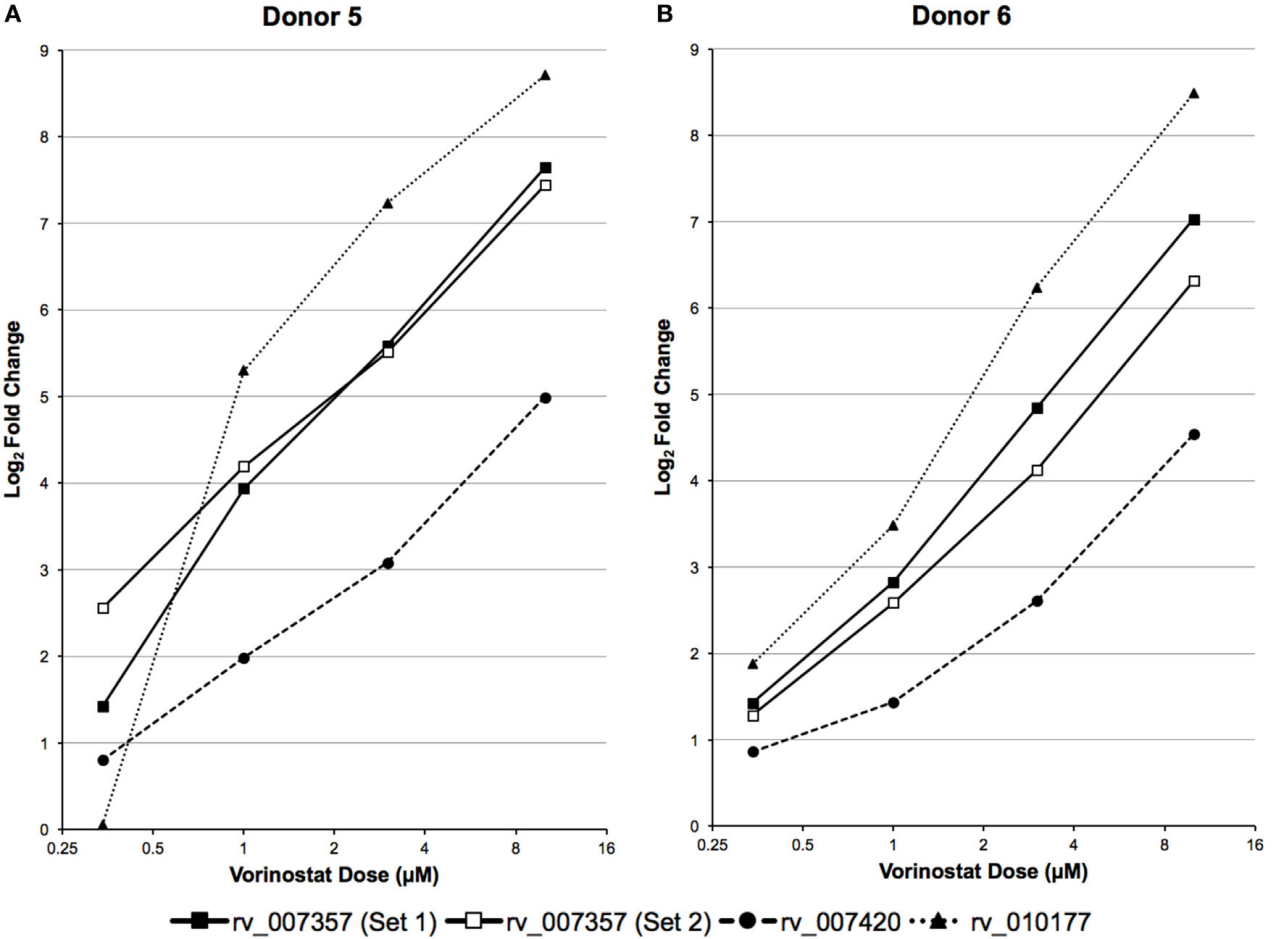
LTR12 elements are upregulated by vorinostat in a dose responsive manner. LTR12 expression was measured by droplet digital PCR using log_2_ fold changes between each dose of vorinostat (0.34, 0.1, 0.3, and 10 μM) and the untreated control as calculated for **(A)** Donor 5 and **(B)** Donor 6. The labels “Set 1” and “Set 2” indicate the two distinct primer and probe sets used to target the same LTR12 element (i.e., rv_007357). LTR12 elements are labeled with their “rv” designation according to the Human Endogenous Retrovirus Database.

A high dose of vorinostat (10 µM) was used to treat primary CD4^+^ T cells subjected to RNA-Seq analysis. This dose is just above what can be achieved with intravenous administration of vorinostat for the treatment of B- and T-cell malignancies (36) and much higher than doses achieved via oral administration in clinical trials to explore “Shock and Kill” strategies for an HIV cure (9, 10). Furthermore, 10 µM of vorinostat appears cytotoxic for just over 20% of healthy T lymphocytes (37). Therefore, the expression of the three LTR12 elements was examined by ddPCR over a more pharmacologically relevant dose–response curve (0.34, 1, 3, and 10 µM), which included less cytotoxic doses (e.g., 0.34 and 1 µM), in primary CD4^+^ T cells isolated from two independent donors. The expression of each LTR12 element was clearly dose dependent for each donor and greater than twofold (log_2_ fold change > 1) for rv_007357 at the lowest dose of 0.34 µM for both donors (Figure 4). In summary, vorinostat upregulated LTR12 elements at doses used to treat blood cell malignancies (36) and at even lower doses (i.e., 0.34 µM) that are relevant to clinical trials with HIV-infected individuals (9, 10).

## Discussion

A total RNA-Seq experiment was used to investigate the modulation of HERVs in primary CD4^+^ T cells exposed to vorinostat. The primary result from this work demonstrated the power of such an untargeted (i.e., unbiased) approach. Our previous studies, using a targeted RT-qPCR approach, had concluded that vorinostat did not modulate HERV elements of the HERV-K, HERV-W, and HERV-FRD families (14). Although this result was confirmed for elements from these families (Figure S1 in Supplementary Material), the current untargeted approach suggests that vorinostat does indeed modulate HERV elements in primary CD4^+^ T cells, predominantly LTRs (e.g., LTR12, LTR16C, and LTR33) of the ERV9 and ERVL families (Figure 1). Brocks and coworkers (38) recently used Cap Analysis Gene Expression Sequencing (CAGE-Seq) (39) to identify expression from transcription start sites (TSS) in a lung cancer cell line (NCI-1299) exposed to vorinostat. The CAGE-Seq data were used to identify treatment induced non-annotated TSS, which were shown to be enriched for LTR12 elements, thus confirming the expression of these elements by vorinostat in a cell line using a different methodology. Furthermore, Kronung and colleagues (20) noted that vorinostat could activate the expression of LTR12-driven genes (e.g., *TP63* and *TNFRSF10B*) in cell lines (i.e., J-LAT8.4 and U1) but did not modulate the expression of HERV-E, HERV-H or MaLR-driven genes, and thus concluded that vorinostat was specific for ERV9 LTRs (i.e., LTR12). However, the current study suggests that vorinostat modulates the expression of LTRs outside of the ERV9 family (e.g., LTR16C and LTR33). In summary, these results advocate for untargeted approaches (e.g., total RNA-Seq and CAGE-Seq), often referred to as “fishing expeditions,” since targeted approaches may not interrogate all relevant transcripts.

The ERV9 family, which includes the LTR12 element, is one of the most successful inhabitants of the human genome due to its continued proliferation until almost six million years ago, around the time of the human-chimp split (40). In contrast, the ERVL family is probably the oldest family and appears to lack an *env* gene consistent with these elements being ancient retrotransposons that entered genomes before the mammalian radiation (41). It is not clear why the transcription of both of these HERV families is modulated by an HDAC inhibitor (i.e., vorinostat) but suggests that HDACs may be an important epigenetic checkpoint in their transcription. HERV elements have recently been associated with non-coding regulatory RNAs with diverse properties ranging from contributing to the pluripotency of human cells (42) to promoting immunoglobulin M production in B-cell driven immune responses independent of T-cells (43). Therefore, agents such as vorinostat that alter the function of HDACs may need to undergo additional evaluation for HERV upregulation to assess their impact on the function of immune cells.

Modulation of HERV elements by vorinostat may be considered an “off-target” effect with respect to the primary goal of HIV activation for shock and kill strategies to facilitate a cure (7). A limitation of this study is that the pathological consequences of these off-target effects remain unknown (16), but one concern might be the oncogenic effects of LTR-driven genes modulated by vorinostat. Lamprecht et al. (44) have demonstrated that activation of an LTR of the MaLR family drives the expression of a proto-oncogene (i.e., *CSF1R*) that may lead to the development of Hodgkin lymphoma. However, vorinostat appears to drive the expression of pro-apoptotic genes (i.e., *TP3* and *TNFRSF10B*) in cell lines and thus protect against tumorigenesis (20). An alternate view would be that the HERV elements modulated by vorinostat encode products that might facilitate HIV activation. To explore this more fully, future analyses should better characterize the transcripts expressed from HERV elements upregulated by vorinostat treatment with respect to the relevant RNA products (e.g., messenger RNA, long non-coding RNA or micro RNA) and determine their role, if any, in HIV activation.

Another potential oncogenic concern would be that replication-defective HERV elements could recombine to form replication-competent virus with tumor inducing potential. This phenomenon has been observed *in vivo* in mice (45–48). Specifically, Young et al. (45) demonstrated that a series of recombination events could restore a replication-defective ERV (i.e., Emv2) to a replication competent virus in antibody-deficient mice (Rag1−/−) that eventually led to thymic and splenic tumors. In humans, the most recently integrated HERV elements belong to the HML-2 family of the HERV-K group, known as HERV-K(HML-2), which have maintained open reading frames encoding functional viral proteins that are expressed but form non-infectious particles (49–52). Dewwaniux et al. (53) demonstrated that the human genome still has the coding potential to resurrect infectious retroviruses from replication defective HERV-K(HML-2) elements. However, this required a three-fragment recombination event *in vitro*, and such a resurrected virus has not been observed to our knowledge *in vivo* in humans. In summary, despite these observations, it is highly unlikely that the HERV elements upregulated by vorinostat in this study, which are predominantly LTR fragments from older HERV families, could recombine to reconstitute the full-length genome required to generate an infectious element.

A final concern is that vorinostat activation of both HIV and HERV elements may lead to recombination and the evolution of novel retroviruses with unknown pathogenicity (19). Acute HIV infection has been shown to lead to the activation of HERV-K(HML-2) elements (22, 54), probably mediated through interactions with the Tat protein. Although subsequent intra-HERV-K recombination has been suggested (55), this remains unconfirmed by other groups (22). Furthermore, to the best of our knowledge, such a recombination event between distantly related retroviruses, such as HERVs and HIV, has never been described. It is unlikely that the HERV elements upregulated in this study by vorinostat exhibit sufficient similarity to facilitate efficient homologous recombination with the HIV genome (56). For example, the LTR12 elements examined in this study exhibit limited similarity with the HIV LTR from the HXB2 strain (accession number K03455): rv_007357 (38%), rv_007420 (23%), and rv_010177 (10%). A limitation of this study is that HERV element modulation by vorinostat was conducted in the absence of HIV infection. HIV infection was excluded in this pilot investigational study because the virus itself can modulate HERV element expression (21, 22), and this confounder was removed so that the effects of HDAC inhibition on HERV element expression could be unambiguously assessed. Future studies of vorinostat treatment of HIV-infected cells could screen for HIV:HERV recombinants, although these are unlikely to be found for the reasons stated earlier.

Analysis of total RNA-Seq data and validation by ddPCR primarily focused on HERV elements upregulated by vorinostat, i.e., LTR12. A potential limitation is that HERV elements may be embedded or in close proximity to genes that are upregulated, and their signal results from read through transcription. This was not likely for the three LTR12 elements selected for ddPCR confirmation (Figures 3 and 4), since they were at least 5 kbp from protein coding genes in the genome. In addition, a large number of HERV elements were also downregulated (e.g., ERVL, Figure 1). This is reflective of our previous work examining vorinostat-modulated gene expression in which the number of genes downregulated was similar to the number upregulated (2–4). In a similar vein, vorinostat leads to chromatin relaxation and then the milieu of transcription factors present in the nucleus regulates which HERV elements are upregulated and which are downregulated due to the corresponding transcription factor binding sites in these elements. Specifically, we have previously determined that vorinostat upregulated the high mobility group (HMG) AT-hook 1 (*HMGA1*) transcription factor at the transcript, protein, and acetylation level (4).

In summary, the modulation of a large number of HERV elements by vorinostat was demonstrated using an unbiased approach (i.e., RNA-Seq). Evidence for the pathogenic consequences of HERV modulation is not of sufficient strength to limit the use of vorinostat in shock and kill approaches toward an HIV cure. However, HERV elements such as LTR12 could be monitored as off-target biomarkers during shock and kill clinical trials with HDAC inhibitors and trial subjects should be screened to further explore HIV:HERV interactions.

## Ethics Statement

This study was carried out in accordance with the recommendations of the Institutional Review Board (IRB) at the University of California San Diego (UCSD) with written informed consent from all subjects who donated blood for this study. All subjects gave written informed consent in accordance with the Declaration of Helsinki. The protocol was approved by the IRB at UCSD.

## Author Contributions

CWh, NB-B, and CWo contributed to the design of the work. NB-B and SL were responsible for the acquisition of the data which was analyzed by CWh, NB-B, SL, and MB. All the authors contributed to the interpretation of the data. CWh, NB-B, and CWo were primarily responsible for drafting the manuscript with significant inputs from TH and GM with edits and revisions suggested by all other authors.

## Conflict of Interest Statement

The senior author, CWo, is currently employed by Merck & Co. who manufactures vorinostat but the research presented in this manuscript was completed prior to this appointment while still a professor at the University of Southampton. The other authors declare that the research was conducted in the absence of any commercial or financial relationships that could be construed as a potential conflict of interest.
