# Supplementary material for: Transcriptional Modulation of Human Endogenous Retroviruses in Primary CD4+ T Cells Following Vorinostat Treatment
Source: Front Immunol. 2018 Apr 12;9:603. doi: 10.3389/fimmu.2018.00603 (PMC5906534; doi:10.3389/fimmu.2018.00603)
Supplement: Supplementary file 1 [file image_1.PDF]

SUPPLEMENTAL FIGURES

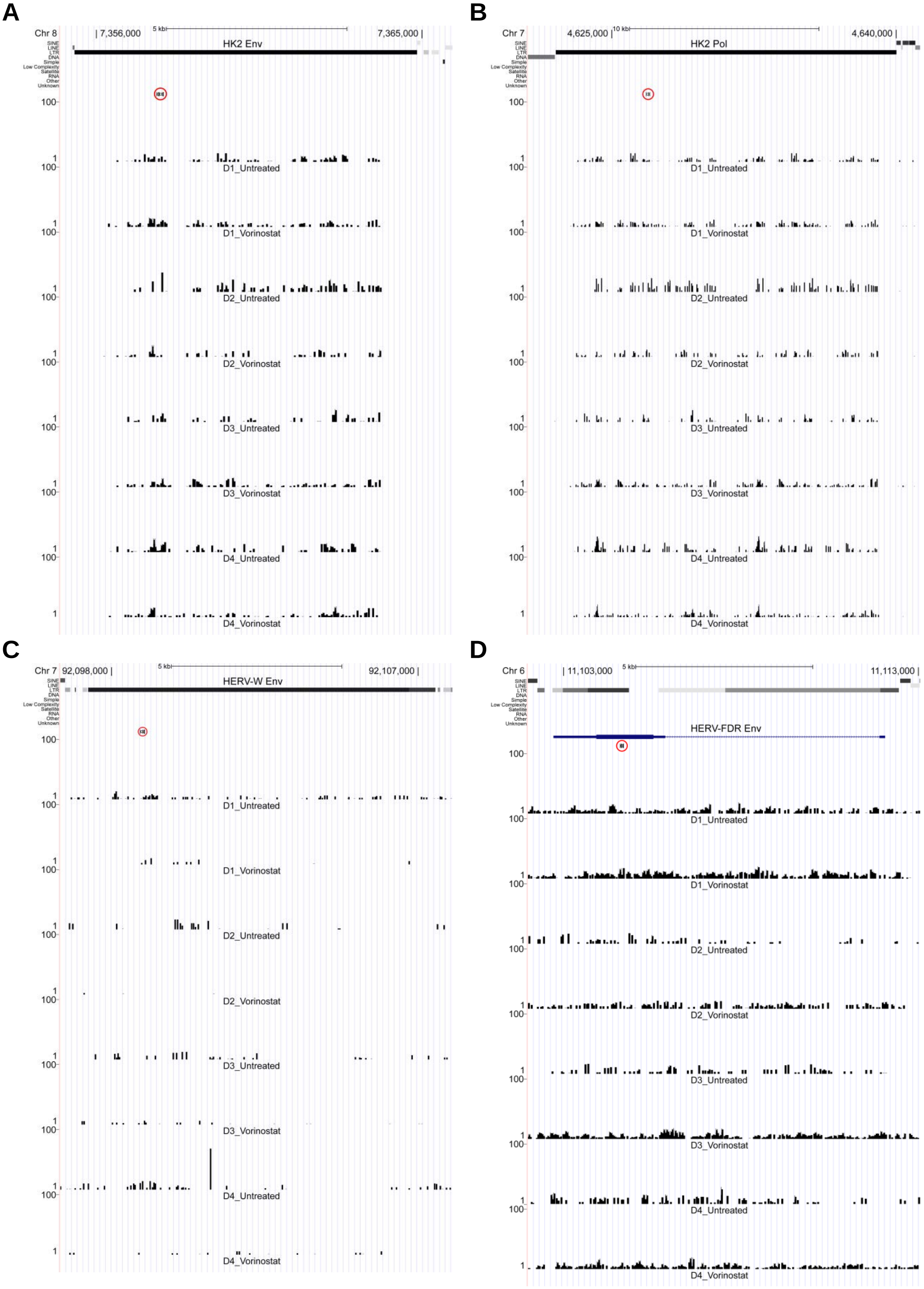

Supplemental Figure 1. **Expression of HK2, HERV-W and HERV-FRD elements visualized as read pile up using the UCSC genome browser.** Reads from the total RNA-Seq experiment were mapped to the human genome and then uploaded to the UCSC genome browser for visualization for each HERV element: (**A**) HK2 *env*, (**B**) HK2 *pol*, (**C**) HERV-W *env*, and (**D**) HERV-FRD *env*. Read tracts are labelled with the donor and condition, e.g. *D1* for Donor 1, *Vorinostat* for drug treated, and *Untreated* for the untreated control (i.e. DMSO solvent alone). Chromosomal coordinates are depicted at the top of each figure and the black (A, B, and C) or blue (D) bar below indicates the position of the HERV element. The y-axis indicates the read level averaged over 40 bp and red circles indicate the location of primers and probe sets used for RT-qPCR detection of these elements in Hurst *et al.* 2016.
